# Hox Genes in Cardiovascular Development and Diseases

**DOI:** 10.3390/jdb4020014

**Published:** 2016-03-24

**Authors:** Marine Roux, Stéphane Zaffran

**Affiliations:** 1 Aix Marseille Université, GMGF UMRS910, Faculté de Médecine, 27 Bd Jean Moulin, Marseille 13385, France; 2 Inserm U910, Faculté de Médecine, 27 Bd Jean Moulin, Marseille 13005, France; 3 Laboratory of Genetics and Development, Institut de Recherches Cliniques de Montréal (IRCM), Université de Montréal, Montréal, QC H3C 3T5, Canada; marine.roux@ircm.qc.ca

**Keywords:** Hox, heart development, cardiogenesis, second heart field, pharyngeal aortic arches, transcription factors

## Abstract

Congenital heart defects (CHD) are the leading cause of death in the first year of life. Over the past 20 years, much effort has been focused on unraveling the genetic bases of CHD. In particular, studies in human genetics coupled with those of model organisms have provided valuable insights into the gene regulatory networks underlying CHD pathogenesis. *Hox* genes encode transcription factors that are required for the patterning of the anterior–posterior axis in the embryo. In this review, we focus on the emerging role of anteriorly expressed *Hox* genes (*Hoxa1*, *Hoxb1*, and *Hoxa3*) in cardiac development, specifically their contribution to patterning of cardiac progenitor cells and formation of the great arteries. Recent evidence regarding the cooperative regulation of heart development by Hox proteins with members of the TALE-class of homeodomain proteins such as Pbx and Meis transcription factors is also discussed. These findings are highly relevant to human pathologies as they pinpoint new genes that increase susceptibility to cardiac anomalies and provide novel mechanistic insights into CHD.

## 1. Introduction

In vertebrates, the roles of the cardiovascular system, consisting of the heart and blood vessels, are diverse and include important functions such as the transport of nutrients and waste, and the distribution of oxygen during both fetal and post-natal life. Cardiovascular development is a complex but ordered process that is spatially and temporally regulated [1]. This process includes dynamic remodeling of the pharyngeal arch arteries (PAAs) and coordinated septation of the outflow tract (OFT). Hence, a slight perturbation in this process may result in a spectrum of cardiovascular defects. In humans, variably abnormal cardiovascular development is reflected in the high incidence of congenital heart diseases (CHD), affecting nearly 1%–2% of all live births [2]. The cause of CHD is often difficult to determine with certainty; nonetheless, studying factors that control cardiovascular development can help us better understand the etiology of these defects. For a detailed summary of many other molecular pathways guiding heart formation, we suggest reviews [3,4,5,6,7].

**Figure 1:**
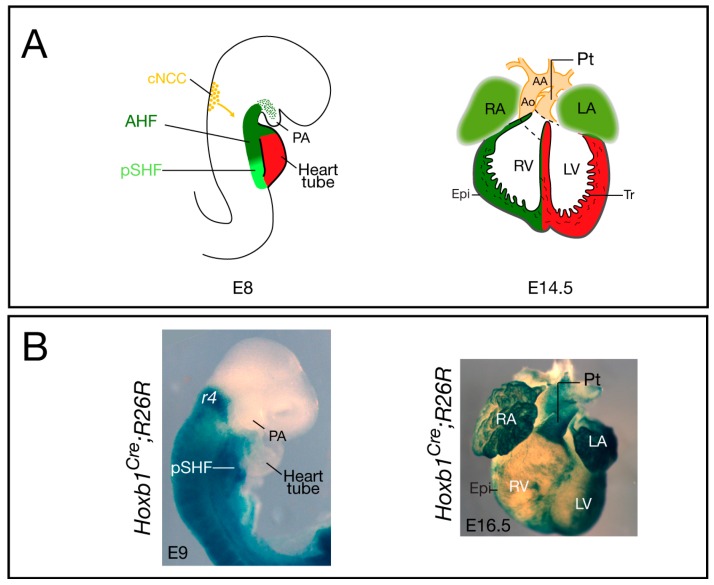
Cardiovascular development and contribution of *Hoxb1^Cre^*-labeled cells in the mouse. (**A**) The location and contribution of the SHF is shown in green, with the anterior heart field (AHF) subdomain in dark green and the posterior SHF (pSHF) in light green. Cardiac neural crest cells (cNCC) are in orange. At E8, the early cardiac tube forms at the midline and it subsequently undergoes looping; at E14.5, the chambers are separated by the inter-ventricular septum and are connected to the pulmonary trunk (Pt) and aorta (Ao); (**B**) Cre lineage visualized by X-gal staining of *Hoxb1^Cre^*; *R26R-lacZ* embryo (lateral view), and heart (ventral view), respectively. At E9, X-gal staining shows *Hoxb1^cre^*+ cells in the pSHF; at E16.5, β-galactosidase activity is detected in both atria and the sub-pulmonary myocardium of *Hoxb1^Cre^*; *R26R-lacZ* heart. *Hoxb1^cre^*+ cells also contribute to the epicardium (Epi), which is located at the surface of the heart. AA: Aortic arch; LA: Left atrium; LV: Left ventricle; PA: Pharyngeal arch; RA: Right atrium; RV: Right ventricle; Tr: Trabeculae.

Embryonic heart development begins at the cardiac crescent stage, around embryonic day (E) 7.5 in the mouse, when differentiation of cardiac precursors to cardiomyocytes is first observed. The linear heart tube that is subsequently formed is a transient structure composed of an inner layer, the endocardium, and covered by a myocardial layer. The heart tube rapidly elongates by progressive addition of cells at the arterial and venous poles during looping morphogenesis. The source of these new cardiomyocytes is located in a region of pharyngeal mesoderm termed the second heart field (SHF) [5,6,7]. As SHF progenitor cells are added to the forming heart, they are exposed to a number of surrounding signals [5,7]. After rightward looping, the heart is shaped by expansion of the myocardium, which leads to the formation of four cardiac chambers, two atria and two ventricles (Figure 1). The forming heart is connected to the bilateral dorsal aorta by the aortic sac and the PAAs. Septation of the cardiac chambers and the OFT is required for separation of the oxygenated and carbonated blood flows. Simultaneous development of the great arteries that supply the head, neck, and upper limbs is also crucial for the developing embryo. This process involves extensive vascular remodeling of the five pharyngeal arch arteries to form a unique arterial architecture (Figure 1A). Although initially symmetric, the most rostral PAAs in the 1st and 2nd arches mostly regress, whereas the caudal PAAs are remodeled into adult vascular structures. Development of the OFT and PAAs requires a specific subpopulation of neural crest cells (NCCs) originating in the posterior hindbrain. These cardiac NCCs migrate to the pharyngeal arches and heart to regulate the remodeling of the PAAs and the septation of the OFT, respectively [8]. They remain as smooth muscle cells of the proximal aorta, carotid arteries, and jugular veins [9].

*Hox* genes encode for highly conserved homeodomain transcription factors. During vertebrate development, Hox proteins establish positional identity along the anterior–posterior (A–P) axis [10]. In mammals, *Hox* genes are organized in analogous clusters on four distinct chromosomes and are expressed in defined and often overlapping domains along the body axis in a manner corresponding to their position along the chromosome. This propriety is known as spatial collinearity [11]. Hox genes also show temporal collinearity, meaning that genes expressed more rostrally are produced before those at the caudal end. Major signaling pathways, such as Fibroblast growth factors (Fgf), retinoic acid (RA), and Wnt, play important roles in the establishment of ordered domains of *Hox* expression. In particular, exogenous RA can induce anterior expansions of *Hox* expression domains. Consistently, several *Hox* genes have well characterized RA-response elements (RAREs) in their enhancers or proximal promoters [12].

Several studies clearly identified that *Hox* function is required during heart development. In the invertebrate *Drosophila melanogaster*, four *Hox* genes are expressed in the organ equivalent of the heart, the dorsal vessel, in essentially non-overlapping domains: *Antennapedia* (*Antp*, from segment T3 to A1), *Ultrabithorax* (*Ubx*, from segment A1 to A4), *abdominal-A* (*abdA*, from segment A4 to A7) and *Abdominal-B* (*AbdB*, the last pair of cardiomyocytes of A7) [13,14]. Furthermore, functional experiments have demonstrated that these genes play critical roles in multiple phases of heart development in *Drosophila*: in the choice of cell lineages within the cardiac mesoderm, in the differentiation of cardiac cells, and in the remodeling of the larval cardiac tube into the adult heart [13,14]. A role for *Hox* gene expression in the vertebrate heart was first suggested by evidence that vitamin A deficiency causes various cardiac anomalies [15]. Vitamin A is the requisite source of RA in the body. Expression of *hoxd-3, hoxa-4*, and *hoxd-4* genes is increased in chick cardiac explants upon RA treatment [16]. In zebrafish, injection of a hyperactive VP16-tagged *hoxb5b* mRNA caused severe cardiac reduction, a highly reminiscent effect of ectopic RA on the formation of atrial and ventricular chambers [17].

In this review we will discuss studies establishing the role of *Hox* genes in several aspects of cardiovascular system development in mammals, from early cardiac specification and heart patterning to the remodeling of the PAAs. Recent studies reported a novel role of the *Hox* genes in the SHF related to regulation by RA signaling. This population of cardiac progenitor cells contributes to the major components of the heart including cardiac OFT, right ventricular, and atrial myocardium. Therefore, altering this early role of the *Hox* genes has later consequences on the onset of malformations of the heart and great arteries such as those described in CHD.

## 2. *Hox* Genes and Lineage Commitment

During gastrulation, the cardiac progenitors are derivatives of the mesoderm, which emerges from the primitive streak. Lineage tracing experiments in the mouse have shown that the first mesodermal cell lineage to emerge is Flk1+ [18]. At the same time, activation of genes along the Hox clusters occurs sequentially in the primitive streak throughout axis elongation [10]. Several signaling pathways, including BMP, FGF, and Wnt, are required for the specification of cardiogenic mesoderm [19]. A recent study showed that coordination of these pathways induces patterning of the mesoderm and specification by activating Cdx/Hox genes, emphasizing the role of Hox factors during this process [20]. Migration of cells expressing *Mesp1*, the earliest molecular marker for cardiac progenitors, through the primitive streak is triggered by Wnt3a signaling [21]. Interestingly, Lengerke *et al.* [20] demonstrated that addition of Wnt3a to embryonic stem (ES) cells is sufficient to induce expression of posterior *Hox* genes. This was the first study to link Wnt3a to Hox genes. These findings suggest that a Mesp1+ migrating cell that also expressed *Hox* genes may depend on Wnt3a signaling.

How *Hox* gene expression is coordinated during lineage differentiation is still poorly understood. However, long intergenic non-coding RNAs, in addition to Trithorax and Polycomb complexes, have been shown to be important for their regulation [22]. Deng *et al.* [23] have recently reported the role of HoxBlinc in mesoderm specification and *HoxB* gene activation. This study shows that HoxBlinc RNA specifies Flk1+ mesoderm toward cardiac and hematopoietic lineages. It recruits Setd1a/MLL1 complexes and facilitates the organization of a chromatin structure that activates the anterior Hoxb genes to control lineage-specific transcription. This work demonstrated that loss of HoxBlinc RNA leads to a strong decrease in *Hoxb1-b6* gene expression and a downregulation of key transcription factors of cardiac development such as Islet1, Nkx2-5, Mef2c, and Gata4 [23]. Therefore, this study reveals a novel cooperation between lncRNA and *Hox* genes during the specification and differentiation of a specific lineage.

## 3. *Hox* Genes and Patterning of the Second Heart Field

Elongation of the cardiac tube occurs by addition of SHF progenitor cells at the arterial and venous poles of the forming heart. Recent studies have clarified the origin of these cardiac progenitors and their regulation (see [3,5,6,7]). SHF cells are characterized by elevated proliferation and delayed differentiation, and by expression of the transcription factors Islet 1 [24], Nkx2-5 [25], and Tbx1 [26]. Since its discovery, there has been accumulating evidence showing that the SHF is pre-patterned [27,28]. Anterior and posterior SHF populations have been identified that contribute to the formation of OFT and right ventricular myocardium at the arterial pole and a large part of the atrial myocardium at the venous pole, respectively [3] (Figure 1A). However, the factors required for the A–P patterning of the SHF were, until recently, unknown. Analysis of the expression pattern of several *Hox* genes in the early embryo demonstrated that in the mesoderm of the SHF, the most anterior expression domains of *Hoxa1*, *Hoxb1*, and *Hoxa3* overlap with the posterior limit of *Islet1* and *Tbx5* [29]. Genetic lineage tracing confirmed that *Hox*-expressing SHF cells contribute to the heart (Figure 1B). Surprisingly, this study revealed that *Hoxb1^Cre^*-labeled cells contribute to both poles of the heart tube: the inferior wall of the OFT, which gives rise to the sub-pulmonary myocardium at the arterial pole, and the atrio-ventricular canal and atrial myocardium at the venous pole [29]. Similarly, lineage tracing of *Hoxa1-enhIII-Cre*- and *Hoxa3^Cre^*-labeled cells showed contributions of *Hoxa1*- and *Hoxa3*-expressing cells to both the atria and the distal part of the OFT [29,30]. Consistent with these observations, a recent retrospective clonal analysis confirmed that sub-pulmonary and atrial myocardial cells are clonally related [31].

The pre-patterning of the SHF into domains contributing to both the arterial and venous pole of the heart begs the question of how it is established. Studies of several animal models have shown that discrete levels of RA signaling are required for the A–P patterning of the heart tube (see [15]). In the mouse, loss of function of retinaldehyde dehydrogenase 2 (*Raldh2*), which encodes a critical enzyme for the synthesis of endogenous RA in the early embryo, results in an abnormal heart, with hypoplastic inflow tract region [32]. Further investigations of *Raldh2* mutant mouse embryos have shown that RA signaling is required to define the posterior boundary of the SHF [33,34]. Expression domains of a number of SHF genes, including *Islet1*, *Tbx1*, *Fgf8*, and *Fgf10*, are caudally expanded in *Raldh2^−/−^*. Additionally, perturbation of RA signaling causes a failure in the deployment of SHF-derived cells, which contributes to the inferior wall of OFT myocardium [29]. In zebrafish, RA signaling has also been shown to regulate the size of the cardiac field through indirect regulation of *hoxb5b* expression in the adjacent forelimb field [35].

Many *Hox* loci contain RA-response elements that control aspects of their expression [36,37]. Consistently, several novel enhancers containing RA-responsive elements have been identified and characterized in the *HoxA* and *HoxB* gene clusters as well as the *Hoxa3* gene [38,39]. When mouse embryos are treated with a teratogenic dose of trans-RA, an anterior shift of the rostral border of *Hoxa1*, *Hoxb1*, and *Hoxa3* expression domains, including the SHF, is observed [29]. Together, these findings suggest that *Hox* gene expression is sensitive to RA dosage and that the influence of RA on heart development may be mediated through its effects on *Hox* genes.

Perturbation of the SHF during elongation of the forming heart tube results in a spectrum of conotruncal congenital defects, ranging from OFT misalignment, including double outlet right ventricle (DORV), overriding aorta, and tetralogy of Fallot (ToF), to ventricular septal defects (VSD) [40]. Several studies showed that human patients carrying a homozygous truncating mutation in *HOXA1* have OFT malformations [41,42]. Interestingly, similar defects have recently been reported for *Hoxa1*-deficient mice (Table 1), demonstrating that *Hoxa1* is required for patterning of the OFT of the heart [43]. More recently, we identified a previously unknown role for the *Hoxb1* gene in the forming heart [44]. Mouse embryos carrying a single *Hoxb1* null allele have VSD and OFT defects (Table 1). These malformations are the consequence of a failure in the deployment of SHF cells during the formation of the OFT [44]. Interestingly, it is the balance between proliferation and differentiation in the SHF that is disrupted in embryos lacking *Hoxb1*. *Hox* genes might also play a role in the differentiation of cardiac progenitor cells. Indeed, reduction of the length of the OFT and increased incidence of heart defects were observed in compound *Hoxa1^−/−^*;*Hoxb1^+/−^* embryos compared to *Hoxa1^−/−^* mutants, demonstrating an overlap in function between these two genes during the formation of the OFT [44]. In 2015, Dupays *et al.* [45] reassessed the expression pattern of *Meis1* gene, which encodes a member of the TALE-class factors in the early embryo, and showed that it is strongly expressed in both the anterior region of the SHF and in the distal part of the OFT. Targeted mutations in the *Meis1* locus lead to early lethality and cardiac anomalies including OFT defects [46,47]. Altogether these data suggest that anterior Hox proteins, probably with cofactors, are important for the formation of the OFT through the development of the SHF.

## 4. Role of *Hox* Genes in Pharyngeal Arch Development and Patterning

During development, PAAs form asymmetrically, following a rostro-caudal and temporal gradient [3]. Remodeling begins around E11.5 when the blood flow at the level of the left 6th arch increases and as the 1st and 2nd arch arteries have already regressed. This results from the rotation of the myocardium and the preferential development of the aorta on the left side to the detriment of the right side [58]. Segments of the dorsal aorta will then regress, allowing individualization of the future common carotid arteries, derived from the 3rd arches. At E14.5, the left 4th arch contributes to the segment of the aortic arch between the carotid and left subclavian arteries, whereas the right one forms a segment connecting the right subclavian arteries to the arterial brachiocephalic trunk. Finally, the right 6th arch is not maintained when the left contributes to the ductus arteriosus that will be shut at birth to allow the establishment of the pulmonary circulation. Anomalies in the patterning or the remodeling of the PAAs, especially of the 4th arch, are critical in the outcome of aortic arch defects such as interruption of the aortic arch (IAA) or subclavian anomalies. NCCs have been shown to be essential for this response.

The NCCs represent a population of multipotent cells with high migratory capacity that will differentiate into a wide range of cell types [59]. A subpopulation of cranial NCCs located in the neural plate between the otic placode and the third pair of somites has been renamed the “cardiac neural crest” for its important contribution to heart development [8,60]. However, a wider subpopulation participates in vascular remodeling between the heart and the face [9]. NCCs migrate through the pharyngeal (also called branchial) arches 3, 4, and 6 and invade the heart by the arterial pole. They contribute to the smooth muscles of the arteries, to the OFT endocardial cushions as well as to the aortico-pulmonary septum separating the aorta and the pulmonary trunk [8,59]. Genetic lineage tracing of NCCs using the *Wnt1^Cre^* mouse revealed their contribution to ascending aorta smooth muscle, to the aortic arch, proximal carotid, and coronary arteries [61]. The first cells invading the OFT participate to the formation of the aortico-pulmonary septum and those migrating later contribute to the remodeling of the PAAs, in particular to the smooth muscle wall of the arteries themselves [62].

Much of our early knowledge about the role of cardiac neural crest in heart development comes from chick ablation studies [8]. Mechanical ablation of premigratory NCCs leads to a number of cardiovascular and non-cardiovascular defects. In the neural crest-ablated embryos, the PAAs are abnormally patterned. When cardiac NCCs are ablated, the PAAs form normally but regress or persist in an unpredictable manner [63,64]. These results suggest that NCCs are not required for the initial formation of the endothelial channels that form the PAAs, but they are required for their remodeling and maintenance.

Cardiac NCCs express a specific set of *Hox* genes, depending on their rostro-caudal origin. Expression of the *Hox* genes in the posterior hindbrain undergoes a spatial and temporal collinear regulation by RA. Experiments in the chick embryo showed that *Hoxa1* and *Hoxb1* participate in neural crest specification and epithelial-to-mesenchymal transition by interacting with neural crest-inducing signaling pathways and regulating the expression of key genes involved in these processes, *Snail2* and *Msx1/2* [65]. They also contribute to the establishment and maintaining of *Hoxb1* expression in NCCs derived from rhombomere 4 [66].

The first model showing abnormal great arteries in absence of Hox function was a knockout mouse model for *Hoxa3* with common carotid artery patterning and remodeling defects [50,52]. NCCs invading the third pharyngeal arch normally express *Hoxa3*. Consistently, the use of a transgenic mouse line *Hoxa3-lacZ* revealed reporter expression in the cardiac NCCs [38]. In *Hoxa3* homozygous null mutant mice, the third arch artery degenerates bilaterally at E11.5, resulting in the malformation of the carotid artery system (Table 1) [51].

*HOXA1* plays a direct role in human cardiovascular system development. Patients with Bosley–Salih–Alorainy syndrome (BSAS) have homozygous truncating mutations in the *HOXA1* locus and exhibit cardiac and cerebrovascular malformations [42,67]. The cardiovascular malformations included interrupted aortic arch type B (IAA-B), aberrant subclavian artery, VSD, and ToF. Genetic lineage tracing analysis in mice shows that *Hoxa1* is expressed in the NCCs that contribute to OFT cushions [29]. Two studies have also reported heart defects including VSD in *Hoxa1* mutant mice, which is coherent with an early role of *Hoxa1* in the NCCs lineage [43,44]. In 2012, Makki and Cappechi [43] demonstrated that *Hoxa1* is required for NCCs specification by acting upstream of the *Zic1* and *Foxd3* transcription factors, two major players in neural crest development.

## 5. Hox Gene Specificity: Cofactors in Heart Development

Hox functions are versatile and their activity seems to be context-dependent. Several examples show that the same Hox can act as an activator or a repressor depending on the circumstances [68]. The high degree of conservation of the homeodomain results in poor sequence specificity, which can be improved through binding with different cofactors. An example is provided by the Hox and TALE (three amino acid loop extension) families [69]. Some Hox interact with the Extradenticle/Pbx family of proteins, whereas others interact preferentially with the Homothorax/Meis family members. Hox proteins also present variable sequences outside the homeodomain, allowing them to interact with an important combination of cofactors. The fact that two factors use the same interaction site, such that only one of the factors can be bound at a time, suggests that there may exist several “Hox complexes,” which vary in subunit composition [70], and most likely in function. Moreover, a recent study found that Hox proteins operate as tissue-specific factors to modulate the ground state binding of TALE cofactors to instruct anatomic identity [71].

In vertebrates, there are four *Pbx* (pre B cell leukemia homeobox) and three *Meis* (myeloid ecotropic viral integration site 1 homolog) genes. PBX proteins also bind to other factors such as HDACs (Histone deacetylase) and HATs (Histone acetyltransferase), suggesting a role in the regulation of the complex activity. A number of groups have reported on the formation of trimeric complexes involving proteins of the HOX, PBX, and MEIS families [72]. Such interaction is likely to modulate the transcriptional activity of the Hox complex [70]. HOX:PBX binds to HDACs, acting as a repressor of transcription that can be reversed by introducing the MEIS protein [73]. Thus, MEIS proteins compete with HDACs for binding to PBX and modulate accessibility of HDACs. Meis1 is able to interact with the co-activator of transcription CBP, which acts as an acetyltransferase, favoring an open position of the chromatin and therefore transcription. HDACs, which interact with PBX, on the contrary, act as a co-repressor.

Recently, Choe and colleagues [74] examined *hoxb1a* regulation in zebrafish embryos and showed that Pbx/Meis association occurs on its promoter but is not enough for transcriptional activation. Recruitment of hoxb1b releases “poised” RNA-PolII, allowing transcription to start. Thus, the authors suggest that TALE factors access promoters during early embryogenesis to poise them for activation but that Hox proteins are required to trigger efficient transcription.

Recent studies have involved Pbx and their Meis partners as critical regulators of cardiovascular development [54,55,57,69,75,76]. *Pbx1* homozygous mutants show great vessel anomalies as well as OFT septation defects [75]. This was confirmed by another study, which also showed a genetic interaction between *Pbx1, Pbx2*, and *Pbx3*, where combinatorial mutants for these genes had additional cardiac malformations [47]. Nevertheless, *Pbx1* displayed the most severe phenotype, with PTA (persistent truncus arteriosus) (Table 1). These studies showed that Pbx proteins act in NCCs to promote Pax3 expression, which is necessary for OFT development [75]. Recent study in zebrafish demonstrated that *pbx4* is required for establishing a proper OFT, probably through the formation of the SHF [54]. Interestingly, *Meis1* disruption results in cardiac anomalies that resemble *Pbx* mutations [47]. This result led the authors to hypothesize that Pbx1 interacts with Hox and/or Meis1 proteins to control a subset of target genes important for OFT development. Meis1 has also been described as a key factor for proliferation of postnatal cardiomyocytes [55]. It remains to be elucidated what genes are direct targets of Pbx/Meis factors.

As for the other Meis proteins, Machon *et al.* [57] showed that *Meis2* is expressed in the NCCs and that its absence results in abnormal OFT development. Heart defects have also been described in a conditional deletion using a NCC-specific Cre; however, no PTAs were observed, suggesting a role for non-crest Meis2-expressing cells in OFT formation (Table 1). Recently, a number of studies reported *MEIS2* mutations in patients with cardiac defects such as VSD [77,78]. In 2012, Paige and colleagues [56] have studied the temporal chromatin alterations in human ES cells as these cells differentiate into cardiomyocytes, and provided a model to determine key regulator genes of heart development. This work characterized those “key” genes as tightly regulated since the consequence of their inappropriate expression could lead to extremely severe defects caused by cell-fate change. *MEIS2* was among the genes identified. Using the zebrafish model, these authors demonstrated that *MEIS2* is critical for cardiac development. Indeed, embryos that received splice-blocking morpholino oligonucleotide directed against *meis2b* had defective cardiac morphogenesis [56].

Despite these studies, the function of TALE factors in cardiovascular development has not yet been fully addressed.

## 6. Conclusions

In this review, we have presented and discussed recent data demonstrating the role of Hox factors and TALE-class members in cardiovascular development. The question of functional redundancy among TALE factors has also been described for *Hoxa1* and *Hoxb1*, which synergize during SHF development [44]. Further investigation involving tissue-specific deletions will help to address this question. In addition, it is likely that Pbx/Meis proteins genetically interact with Hox genes during heart development given the similar phenotypes of *Hoxa1^−/−^*, *Pbx1^−/−^*, and *Meis1^−/−^* mutants. Thus, genetic approaches aimed at deleting one or more alleles of the *Hox*, *Pbx*, and *Meis* genes will provide valuable information regarding the intersection of these genes in OFT development. While recent studies have uncovered the role of Hox and TALE proteins in heart development, little is known about downstream target genes they activate or inhibit. Thus, it will be crucial to identify common target genes, thereby providing insight into potential mechanisms underlying CHDs.

## Figures and Tables

**Table 1:** Cardiac phenotypes associated with *Hox*, *Pbx*, and *Meis* loss-of-function.

Gene	Mutants	Anomalies	References
***Hoxa1***	*Hoxa-1.6^−/−^*	No cardiac phenotype VSD	[48]
	*Hoxa-1.6^−/−^;Hoxb1^GFP/+^*	OFT defects, VSD	[44]
	*Hoxa1^GFPneo/GFPneo^*	No cardiac phenotype	[49]
	*Hoxa1^−/−^*	IAA-B, ASC, RAA, VSD, ToF	[43]
***Hoxa3***	*Hox-1.5^−/−^*	No cardiac phenotype	[50]
	*Hoxa3^−/−^*	Degeneration of the 3rd arch artery Malformation of the carotid artery system	[51,52]
***Hoxb1***	*Hoxb1^GFP/GFP^*	OFT defects, VSD	[44]
***HoxA/HoxB***	*Hoxa^−/−^;Hoxb^−/−^*	Heart looping defects	[53]
***Pbx1***	*Pbx1^−/−^*	Die around E15; PTA and VSD	[47]
	*Pbx1^+/−^;Pbx2^+/−^;Pbx3^+/−^*	Bicuspid aortic valve (BAV)	[47]
	*Pbx1^+/−^;Pbx2^−/−^*	Overriding aorta, VSD, BAV, bicuspid pulmonary valve	[47]
	*Pbx1^+/−^;Pbx2^−/−^;Pbx3^+/−^*	ToF	[47]
***Pbx2***	*Pbx2^−/−^*	No cardiac phenotype	[47]
***Pbx3***	*Pbx3^−/−^*	No cardiac phenotype	[47]
***Pbx4***	*pbx4^b557−/−^* (zebrafish)	OFT defects	[54]
***Meis1***	*Meis1^−/−^*	Overriding aorta, VSD	[47]
	*Meis1^ECFP/ECFP^*	Die around E14; VSD	[46]
	*α-MHC-Cre;Meis1^f/f^*	Increased postnatal cardiomyocyte proliferation	[55]
***Meis2***	*meis2b*-MO (zebrafish)	Heart looping defects	[56]
	*Meis2^−/−^*	Lethality by E13.5-E15; PTA and VSD	[57]
	*AP2α-Cre;Meis2^f/f^*	DORV, abnormal semilunar valves	[57]

## References

[1] Poelmann R.E., Gittenberger-de Groot A.C., Hierck B.P. (2008). The development of the heart and microcirculation: Role of shear stress. Med. Biol. Eng. Comput..

[2] Hoffman J.I., Kaplan S. (2002). The incidence of congenital heart disease. J. Am. Coll. Cardiol..

[3] Vincent S.D., Buckingham M.E. (2010). How to make a heart: The origin and regulation of cardiac progenitor cells. Curr. Top. Dev. Biol..

[4] Srivastava D. (2006). Making or breaking the heart: From lineage determination to morphogenesis. Cell.

[5] Zaffran S., Kelly R.G. (2012). New developments in the second heart field. Differentiation.

[6] Buckingham M., Meilhac S., Zaffran S. (2005). Building the mammalian heart from two sources of myocardial cells. Nat. Rev. Genet..

[7] Rochais F., Mesbah K., Kelly R.G. (2009). Signaling pathways controlling second heart field development. Circ. Res..

[8] Kirby M.L., Gale T.F., Stewart D.E. (1983). Neural crest cells contribute to normal aorticopulmonary septation. Science.

[9] Etchevers H.C., Vincent C., le Douarin N.M., Couly G.F. (2001). The cephalic neural crest provides pericytes and smooth muscle cells to all blood vessels of the face and forebrain. Development.

[10] Deschamps J., van Nes J. (2005). Developmental regulation of the hox genes during axial morphogenesis in the mouse. Development.

[11] Duboule D., Dolle P. (1989). The structural and functional organization of the murine hox gene family resembles that of drosophila homeotic genes. EMBO J..

[12] Alexander T., Nolte C., Krumlauf R. (2009). Hox genes and segmentation of the hindbrain and axial skeleton. Annu. Rev. Cell Dev. Biol..

[13] Lo P.C., Frasch M. (2003). Establishing A-P polarity in the embryonic heart tube. A conserved function of Hox genes in *Drosophila* and vertebrates?. Trends Cardiovasc. Med..

[14] Monier B., Tevy M.F., Perrin L., Capovilla M., Semeriva M. (2007). Downstream of homeotic genes: In the heart of hox function. Fly.

[15] Zaffran S., Niederreither K., Dolle P., Niederreither K. (2015). Retinoic acid signaling and heart development. The Retinoids.

[16] Searcy R.D., Yutzey K.E. (1998). Analysis of Hox gene expression during early avian heart development. Dev. Dyn.

[17] Waxman J.S., Yelon D. (2009). Increased Hox activity mimics the teratogenic effects of excess retinoic acid signaling. Dev. Dyn..

[18] Van Vliet P., Wu S.M., Zaffran S., Puceat M. (2012). Early cardiac development: A view from stem cells to embryos. Cardiovasc. Res..

[19] Zaffran S., Frasch M. (2002). Early signals in cardiac development. Circ. Res..

[20] Lengerke C., Schmitt S., Bowman T.V., Jang I.H., Maouche-Chretien L., McKinney-Freeman S., Davidson A.J., Hammerschmidt M., Rentzsch F., Green J.B. (2008). Bmp and Wnt specify hematopoietic fate by activation of the Cdx-Hox pathway. Cell Stem Cell.

[21] Yue Q., Wagstaff L., Yang X., Weijer C., Munsterberg A. (2008). Wnt3a-mediated chemorepulsion controls movement patterns of cardiac progenitors and requires RhoA function. Development.

[22] Soshnikova N. (2014). Hox genes regulation in vertebrates. Dev. Dyn..

[23] Deng C., Li Y., Zhou L., Cho J., Patel B., Terada N., Li Y., Bungert J., Qiu Y., Huang S. (2016). HoxBlinc RNA recruits Set1/MLL complexes to activate Hox gene expression patterns and mesoderm lineage development. Cell Rep..

[24] Cai C.L., Liang X., Shi Y., Chu P.H., Pfaff S.L., Chen J., Evans S. (2003). Isl1 identifies a cardiac progenitor population that proliferates prior to differentiation and contributes a majority of cells to the heart. Dev. Cell.

[25] Prall O.W., Menon M.K., Solloway M.J., Watanabe Y., Zaffran S., Bajolle F., Biben C., McBride J.J., Robertson B.R., Chaulet H. (2007). An Nkx2–5/Bmp2/Smad1 negative feedback loop controls heart progenitor specification and proliferation. Cell.

[26] Baldini A. (2005). Dissecting contiguous gene defects: Tbx1. Curr. Opin. Genet. Dev..

[27] Galli D., Dominguez J.N., Zaffran S., Munk A., Brown N.A., Buckingham M.E. (2008). Atrial myocardium derives from the posterior region of the second heart field, which acquires left-right identity as Pitx2c is expressed. Development.

[28] Zaffran S., Kelly R.G., Meilhac S.M., Buckingham M.E., Brown N.A. (2004). Right ventricular myocardium derives from the anterior heart field. Circ. Res..

[29] Bertrand N., Roux M., Ryckebusch L., Niederreither K., Dolle P., Moon A., Capecchi M., Zaffran S. (2011). Hox genes define distinct progenitor sub-domains within the second heart field. Dev. Biol..

[30] Makki N., Capecchi M.R. (2010). Hoxa1 lineage tracing indicates a direct role for Hoxa1 in the development of the inner ear, the heart, and the third rhombomere. Dev. Biol..

[31] Lescroart F., Mohun T., Meilhac S.M., Bennett M., Buckingham M. (2012). Lineage tree for the venous pole of the heart: Clonal analysis clarifies controversial genealogy based on genetic tracing. Circ. Res..

[32] Niederreither K., Subbarayan V., Dolle P., Chambon P. (1999). Embryonic retinoic acid synthesis is essential for early mouse post-implantation development. Nat. Genet..

[33] Ryckebusch L., Wang Z., Bertrand N., Lin S.C., Chi X., Schwartz R., Zaffran S., Niederreither K. (2008). Retinoic acid deficiency alters second heart field formation. Proc. Natl. Acad. Sci. USA.

[34] Sirbu I.O., Zhao X., Duester G. (2008). Retinoic acid controls heart anteroposterior patterning by down-regulating Isl1 through the Fgf8 pathway. Dev. Dyn..

[35] Waxman J.S., Keegan B.R., Roberts R.W., Poss K.D., Yelon D. (2008). Hoxb5b acts downstream of retinoic acid signaling in the forelimb field to restrict heart field potential in zebrafish. Dev. Cell.

[36] Duester G. (2008). Retinoic acid synthesis and signaling during early organogenesis. Cell.

[37] Niederreither K., Dolle P., Rosenthal N., Harvey R.P. (2008). Retinoids and heart development. Heart Development.

[38] Diman N.Y., Remacle S., Bertrand N., Picard J.J., Zaffran S., Rezsohazy R. (2011). A retinoic acid responsive Hoxa3 transgene expressed in embryonic pharyngeal endoderm, cardiac neural crest and a subdomain of the second heart field. PLoS One.

[39] Nolte C., Jinks T., Wang X., Martinez Pastor M.T., Krumlauf R. (2013). Shadow enhancers flanking the HoxB cluster direct dynamic hox expression in early heart and endoderm development. Dev. Biol..

[40] Ward C., Stadt H., Hutson M., Kirby M.L. (2005). Ablation of the secondary heart field leads to tetralogy of fallot and pulmonary atresia. Dev. Biol..

[41] Holve S., Friedman B., Hoyme H.E., Tarby T.J., Johnstone S.J., Erickson R.P., Clericuzio C.L., Cunniff C. (2003). Athabascan brainstem dysgenesis syndrome. Am. J. Med. Genet. Part A.

[42] Tischfield M.A., Bosley T.M., Salih M.A., Alorainy I.A., Sener E.C., Nester M.J., Oystreck D.T., Chan W.M., Andrews C., Erickson R.P. (2005). Homozygous hoxa1 mutations disrupt human brainstem, inner ear, cardiovascular and cognitive development. Nat. Genet..

[43] Makki N., Capecchi M.R. (2012). Cardiovascular defects in a mouse model of hoxa1 syndrome. Hum. Mol. Genet..

[44] Roux M., Laforest B., Capecchi M., Bertrand N., Zaffran S. (2015). Hoxb1 regulates proliferation and differentiation of second heart field progenitors in pharyngeal mesoderm and genetically interacts with Hoxa1 during cardiac outflow tract development. Dev. Biol..

[45] Dupays L., Shang C., Wilson R., Kotecha S., Wood S., Towers N., Mohun T. (2015). Sequential binding of MEIS1 and NKX2–5 on the Popdc2 gene: A mechanism for spatiotemporal regulation of enhancers during cardiogenesis. Cell Rep..

[46] Gonzalez-Lazaro M., Rosello-Diez A., Delgado I., Carramolino L., Sanguino M.A., Giovinazzo G., Torres M. (2014). Two new targeted alleles for the comprehensive analysis of Meis1 functions in the mouse. Genesis.

[47] Stankunas K., Shang C., Twu K.Y., Kao S.C., Jenkins N.A., Copeland N.G., Sanyal M., Selleri L., Cleary M.L., Chang C.P. (2008). Pbx/Meis deficiencies demonstrate multigenetic origins of congenital heart disease. Circ. Res..

[48] Lufkin T., Dierich A., LeMeur M., Mark M., Chambon P. (1991). Disruption of the Hox-1.6 homeobox gene results in defects in a region corresponding to its rostral domain of expression. Cell.

[49] Godwin A.R., Stadler H.S., Nakamura K., Capecchi M.R. (1998). Detection of targeted GFP-Hox gene fusions during mouse embryogenesis. Proc. Natl. Acad. Sci. USA.

[50] Chisaka O., Capecchi M.R. (1991). Regionally restricted developmental defects resulting from targeted disruption of the mouse homeobox gene Hox-1.5. Nature.

[51] Chisaka O., Kameda Y. (2005). Hoxa3 regulates the proliferation and differentiation of the third pharyngeal arch mesenchyme in mice. Cell Tissue Res..

[52] Kameda Y., Watari-Goshima N., Nishimaki T., Chisaka O. (2003). Disruption of the hoxa3 homeobox gene results in anomalies of the carotid artery system and the arterial baroreceptors. Cell Tissue Res..

[53] Soshnikova N., Dewaele R., Janvier P., Krumlauf R., Duboule D. (2013). Duplications of Hox gene clusters and the emergence of vertebrates. Dev. Biol..

[54] Kao R.M., Rurik J.G., Farr G.H., Dong X.R., Majesky M.W., Maves L. (2015). Pbx4 is required for the temporal onset of zebrafish myocardial differentiation. J. Dev. Biol..

[55] Mahmoud A.I., Kocabas F., Muralidhar S.A., Kimura W., Koura A.S., Thet S., Porrello E.R., Sadek H.A. (2013). Meis1 regulates postnatal cardiomyocyte cell cycle arrest. Nature.

[56] Paige S.L., Thomas S., Stoick-Cooper C.L., Wang H., Maves L., Sandstrom R., Pabon L., Reinecke H., Pratt G., Keller G. (2012). A temporal chromatin signature in human embryonic stem cells identifies regulators of cardiac development. Cell.

[57] Machon O., Masek J., Machonova O., Krauss S., Kozmik Z. (2015). Meis2 is essential for cranial and cardiac neural crest development. BMC Dev. Biol..

[58] Yashiro K., Shiratori H., Hamada H. (2007). Haemodynamics determined by a genetic programme govern asymmetric development of the aortic arch. Nature.

[59] Le Lievre C.S., le Douarin N.M. (1975). Mesenchymal derivatives of the neural crest: Analysis of chimaeric quail and chick embryos. J. Embryol Exp. Morphol..

[60] Kirby M.L., Waldo K.L. (1995). Neural crest and cardiovascular patterning. Circ. Res..

[61] Jiang X., Choudhary B., Merki E., Chien K.R., Maxson R.E., Sucov H.M. (2002). Normal fate and altered function of the cardiac neural crest cell lineage in retinoic acid receptor mutant embryos. Mech. Dev..

[62] Boot M.J., Gittenberger-De Groot A.C., van Iperen L., Hierck B.P., Poelmann R.E. (2003). Spatiotemporally separated cardiac neural crest subpopulations that target the outflow tract septum and pharyngeal arch arteries. Anat. Rec. Part A Discov. Mol. Cell Evol. Biol..

[63] Waldo K.L., Kumiski D., Kirby M.L. (1996). Cardiac neural crest is essential for the persistence rather than the formation of an arch artery. Dev. Dyn.

[64] Hutson M.R., Kirby M.L. (2007). Model systems for the study of heart development and disease cardiac neural crest and conotruncal malformations. Semin. Cell Dev. Biol..

[65] Gouti M., Briscoe J., Gavalas A. (2011). Anterior Hox genes interact with components of the neural crest specification network to induce neural crest fates. Stem Cells.

[66] Studer M., Gavalas A., Marshall H., Ariza-McNaughton L., Rijli F.M., Chambon P., Krumlauf R. (1998). Genetic interactions between Hoxa1 and Hoxb1 reveal new roles in regulation of early hindbrain patterning. Development.

[67] Bosley T.M., Alorainy I.A., Salih M.A., Aldhalaan H.M., Abu-Amero K.K., Oystreck D.T., Tischfield M.A., Engle E.C., Erickson R.P. (2008). The clinical spectrum of homozygous hoxa1 mutations. Am. J. Med. Genet. Part A.

[68] Krasnow M.A., Saffman E.E., Kornfeld K., Hogness D.S. (1989). Transcriptional activation and repression by ultrabithorax proteins in cultured drosophila cells. Cell.

[69] Moens C.B., Selleri L. (2006). Hox cofactors in vertebrate development. Dev. Biol..

[70] Ladam F., Sagerstrom C.G. (2014). Hox regulation of transcription: More complex(es). Dev. Dyn..

[71] Amin S., Donaldson I.J., Zannino D.A., Hensman J., Rattray M., Losa M., Spitz F., Ladam F., Sagerstrom C., Bobola N. (2015). Hoxa2 selectively enhances meis binding to change a branchial arch ground state. Dev. Cell.

[72] Ferretti E., Marshall H., Popperl H., Maconochie M., Krumlauf R., Blasi F. (2000). Segmental expression of Hoxb2 in r4 requires two separate sites that integrate cooperative interactions between Prep1, Pbx and Hox proteins. Development.

[73] Choe S.K., Lu P., Nakamura M., Lee J., Sagerstrom C.G. (2009). Meis cofactors control HDAC and CBP accessibility at Hox-regulated promoters during zebrafish embryogenesis. Dev. Cell.

[74] Choe S.K., Ladam F., Sagerstrom C.G. (2014). Tale factors poise promoters for activation by Hox proteins. Dev. Cell.

[75] Chang C.P., Stankunas K., Shang C., Kao S.C., Twu K.Y., Cleary M.L. (2008). Pbx1 functions in distinct regulatory networks to pattern the great arteries and cardiac outflow tract. Development.

[76] Maves L., Tyler A., Moens C.B., Tapscott S.J. (2009). Pbx acts with Hand2 in early myocardial differentiation. Dev. Biol..

[77] Crowley M.A., Conlin L.K., Zackai E.H., Deardorff M.A., Thiel B.D., Spinner N.B. (2010). Further evidence for the possible role of MEIS2 in the development of cleft palate and cardiac septum. Am. J. Med. Genet. Part A.

[78] Louw J.J., Corveleyn A., Jia Y., Hens G., Gewillig M., Devriendt K. (2015). MEIS2 involvement in cardiac development, cleft palate, and intellectual disability. Am. J. Med. Genet. Part A.

